# Factors influencing consistent use of bed nets for the control of malaria among children under 5 years in Soroti District, North Eastern Uganda

**DOI:** 10.1186/s12936-022-04396-z

**Published:** 2022-12-02

**Authors:** Anne Ruth Akello, John Paul Byagamy, Samuel Etajak, Charles Stephen Okadhi, Adoke Yeka

**Affiliations:** 1 Department of Public Health, Faculty of Health Sciences, Lira University, Lira, Uganda; 2 Deprtment of Disease Control and Environmental Health, School of Public Health, Makerere University, Kampala, Uganda; 3 Soroti District Local Government, Soroti, Uganda

**Keywords:** Malaria control, Consistent use of bed nets, Children under 5 years, Soroti district, Eastern Uganda

## Abstract

**Background:**

The use of insecticide-treated bed nets has been proven to be effective in reducing malaria transmission in highly endemic areas. Use of long-lasting insecticidal nets (LLINs) has been embraced by many malaria endemic countries. LLINs are up to 95% effective in inhibiting blood feeding, when used consistently even after 7 years. The challenge, however, is enhancing their consistent use, especially by the most vulnerable groups (children under 5 years and pregnant women). The study established factors associated with consistent use of bed nets for malaria control among children under 5 years in Soroti district.

**Methods:**

The study employed a cross-sectional design, with multi-stage sampling of households. A total of 400 households (HH) were sampled and the HH head in each household interviewed. Key informant interviews (KIIs) were conducted with 7 key informants who were knowledgeable on the subject matter. Data analysis was done using SPSS 17.0 at Univariate, Bivariate and Multivariable levels; after entry and cleaning. Key informants’ data were summarized manually; verbatim quotes and text used to reinforce quantitative data in line with objectives.

**Results:**

Only 56.8% of the 690 children under 5 years used bed nets consistently. The factors affecting consistent bed net use were age of the child, their use of bed nets the previous night, occupation of caretaker, respondents’ perceived susceptibility, perceived risk of getting malaria, size and shape of the bed nets. Rectangular nets were difficult to hang daily in huts according to most key informants.

**Conclusion:**

Consistent bed net use among under fives is still below the RBM target of 85% by 2015 and can be enhanced by providing conical bed nets and setting aside a health education programme to emphasize the effectiveness of even one mosquito in spreading malaria at night to the entire household and ability of bed nets to stop transmission better than other methods.

**Supplementary Information:**

The online version contains supplementary material available at 10.1186/s12936-022-04396-z.

## Background

Malaria is one of the most important parasitic diseases in the world that kills approximately 1.5 million people every year. About 90% of all malaria deaths in the world today occur in sub-Saharan Africa (SSA), approximating to one million deaths each year; with most of these deaths occurring in children under 5 years [1–3]. This makes malaria a leading cause of mortality in children under 5 years of age and is responsible for 10% of the overall disease burden, 40% of public health expenditure, 30–50% of inpatient admissions, and up to 50% of outpatient visits [4–6]. The majority of infections in Africa are caused by *Plasmodium falciparum*, the most dangerous of the four human malaria parasites and transmitted by the most effective vector, the female *Anopheles gambiae* mosquito. Malaria is widespread in Africa due to favourable conditions, which makes control difficult [7, 8].

In Uganda, malaria is the leading cause of morbidity and mortality and is responsible for up to 40% of all outpatient visits, 25% of all hospital admissions and 14% of all hospital deaths [9, 10]. The overall malaria-specific mortality is estimated to be between 70,000 and 100,000 child deaths annually in Uganda, a death toll that far exceeds that of HIV/AIDS. Between 10 and 12 million clinical cases of malaria are treated in public health systems alone [11]. Besides, between 33 and 54 per cent of absenteeism either at work or at school is attributable to malaria. Thus, malaria may not only be the leading cause of ill health and death in Uganda but also the leading cause of poverty in the country [12].

The WHO and the Roll back malaria (RBM) movement promote the use ITNs for malaria prevention. The use of ITNs has been associated with a significant reduction in all causes of childhood mortality [1, 4]. There is widespread support to increase ITN coverage among vulnerable populations in Africa. An understanding of the factors influencing use of ITNs is important for the success of this strategy [8, 13]. As a consequence, the proportions of children under five sleeping under ITNs the previous night increased from 10% in 2006 to 47% in 2009 and 60% in 2011 to 90% in 2014 [10, 14].

Other studies elsewhere established that factors that caused variation in bed net use in households included the age of the household head, their occupation, gender and education level [15–28]. Additionally, other studies highlight the effect of bed net size and rectangular shape on its use [29].

There is continuous need for critical analyses of how effective preventive interventions against malaria, such as consistent use of bed nets can be improved so as to protect the vulnerable populations in the country [11]. Such studies continue to contribute to the rapid development of commercial mosquito nets and ITN sector which since has shown exponential growth rates [11].

In 2006, only 22% of children under 5 years slept under any type of net, 13.5% slept under an ever treated net and 10% slept under an ITN [29]. By 2009, these proportions increased: 41% of the children under 5 years were reported to have slept under any net the night before the survey, 33% slept under an ITN while 32% slept under a LLIN [30].

Malaria is a leading cause of morbidity in Soroti district. The proportion of outpatient attendance due to malaria rose from 42.3% in 2009 to 54.2% in 2010, dropping slightly to 51.8% by June 2011 [31]. Majority of those affected are children under 5 years of age. Despite the high prevalence, estimated bed net usage among children under 5 years in the district was low (41%) in 2010 [14] and fell far short of the NMCP target of 80% [32].

There was little information on the factors influencing consistent use of bed nets in Soroti district. That could hamper efforts to improve uptake of such effective interventions because the health department and the community would be acting in darkness. Such sustained efforts were required to prevent the resurgence of malaria from where it was eliminated [33].

The information generated could contribute to achievement of target 8 of MDG 6 (Have halted by 2015 and began to reverse the incidence of malaria and other major diseases) [34]. Further more, several studies have been conducted on the use of ITNs, Knowledge, attitudes and practices (KAPs) that influence prevention and control of malaria in children under 5 years [35–47]. The study, therefore, aimed at determining factors influencing consistent use of bed nets among children under 5 years in Soroti district, North Eastern Uganda.

## Methods

### Study design

This was a population-based cross-sectional household survey that employed both quantitative and qualitative methods of data collection.

### Study area

The study was conducted in Soroti district. Soroti district is located in North-Eastern Uganda, neighbouring Kaberamaido district in the West, Amuria district in the North, Katakwi district in the North-East. In the Southern part of the district lies Kamuli, Kumi, Ngora and Pallisa districts, together with Lake Kyoga. The total land area of the district is 2665 km^2^, of which 84.8% is dry land and 15.2% water bodies. Soroti district is made up of two counties, 10 sub-counties, 50 parishes and 386 villages. The district had a population of 305,900 people in 2011; consisting majorly of the Iteso. There was a reported relatively high burden of Communicable diseases, especially malaria (51.8%), acute respiratory-tract infections (ARTIs) 47.6%, diarrhoeal diseases 38.6% and maternal-child health conditions 29.6% [31]. The economic activities include subsistence farming, small businesses, fishing and animal rearing.

### Study population

The study population consisted of children under 5 years of age in Soroti district. The eligible population was residents who were present in the sampled Households (HHs) in the previous 12 months to the survey. Individual interviews were conducted with eligible heads of HHs or any adult living together with children under 5 years in each household. Qualitative data was obtained from key informants, including the DHO, malaria control focal person, ADHO-Environmental Health, ADHO-MCH, DHE, project officers of Stop Malaria Project, Teso Safe Motherhood Project and World Vision Soroti.

### Recruitment criteria

HHs with at least one child under 5 years of age were included in the survey. The respondents were the heads of HHS or their designated spouse or any member of the household who was aged 18 years and above and also individual heads of HHS who lived with children under 5 years daily for the previous 12 months.

### Sample size determination

The sample size for the study was calculated using Kish Leislie’ formula 1965 [48] for a cross-sectional study, with a 5% additional number to cater for non-response.

$$\begin{aligned} n & = g\frac{{z^{2}_{\alpha /2} pq}}{{d^{2} }} = 2*\frac{{1.96^{2} *0.41*0.59}}{{0.07^{2} }} \\ & = 379.2992 + 5\% \;{\text{Non - response}} \\ \end{aligned}$$

$${\text{n}} = 400{\text{ Respondents}}$$

where Z_α/2_: Z-value corresponding to alpha level of significance of 5%; P: estimated proportion of bed net use among children under 5 years in Uganda. P = 41%, obtained from Uganda Malaria Indicator Survey, 2009. q: 1 − p; d: absolute precision; g: design effect because of multi-stage cluster sampling. A total of 7 key informants were interviewed.

### Sample size

The study involved interviewing 400 heads of HHs and 7 key informants purposively chosen with respect to their positions and technical knowledge on consistent use of bed nets in the district.

### Data collection methods

Quantitative and qualitative methods of data collection were used. Quantitative data was obtained using interviewer administered semi-structured household questionnaires while qualitative data was obtained through key informant interviews with 7 key informants.

### Data collection procedures

For quantitative data collection, research assistants were trained for 2 days on the all the aspects of survey procedures meanwhile, for qualitative data collection, the Principal Investigator (PI) conducted key informant interviews with 7 key informants. A written consent was obtained before conducting Key informant interviews. The interviews were guided by open-ended questions in the KI guide. The PI listened critically and wrote down notes of all responses to the questions asked using short hand.

### Pre-testing of data collection tools

Tools were pre-tested in Arapai parish and appropriate adjustments made to ensure a complete capture of required data. Pre-testing also facilitated transformation of semi-structured questionnaires into structured which eased data entry and analysis.

### Data management and analysis

#### Data entry

The questionnaires were checked and edited by the principal investigator and research assistants during and after the data collection exercise. Data was checked for completeness by filling-up any missing links and reconciling any mismatches. Coding of none pre-coded data was done after the whole data collection exercise. Data in each uniquely identified questionnaire was entered into SPSS computer package by the principal investigator.

#### Quality control

Research Assistants (RAs) were trained for 2 days before data collection commenced. Data collection tools were translated into Ateso, the local dialect to ensure consistency in the way the questions were asked for accuracy of the data collected. A pre-test of the data collection tools was conducted in Arapai parish. That allowed research assistants to practice asking the translated questions to ensure consistency and prompt adjustment of data collection tools. Field editing of data and checking for completeness was done daily by the principal investigator and RAs. Key informants were interviewed by the principal investigator to ensure accurate asking of questions and capture of all responses appropriately. The PI listened critically and wrote down notes of all responses to the questions asked using short hand.

#### Data analysis

Quantitative data was entered into SPSS computer package, cleaned and analyzed using SPSS 17.0. The questionnaire unique identifiers were used to merge data on each child enumerated in each household to form a single data set on which analysis was based. Univariate, Bivariate and multivariable analysis was done. Analysis was limited to only those children aged 1–4 years (determined by outcome variable) who used bed nets for protection against mosquitos so as to eliminate the influence of household bed net access. Also further assessment of respondents’ knowledge about malaria was under taken (Additional file 1).

### Ethical approval and consent to participate

We received ethical approval from the Uganda National Council of Science and Technology (UNCST), through the Makerere University School of Public Health Higher Degrees Research and Ethics Committee (HDREC). All respondents provided written informed consent upon receiving details of the study. All eligible participants voluntarily consented, anonymity of the participants and respondents were kept confidential throughout the study.

## Results

### Socio-demographics characteristics of participants

The age of the respondents ranged from 18 to 72 years with a mean age of 36 years and a modal age of 40 years. The majority of respondents, 221 (56.5%) were males. A majority had primary education level 195 (49.9%), most participants were involved in agriculture 291 (74.4) and the majority were Iteso 303 (77.5) as showed in Table 1.

**Table 1:** Socio-demographic characteristics of respondents (N = 391)

Variable	Frequency, N	Percentage (%)
Age group of respondents (years)		
31–40	142	36.3
21–30	129	33.0
41–50	62	15.9
51–60	30	7.7
18–20	19	4.9
61+	9	2.3
Gender		
Male	221	56.5
Female	170	43.5
Education level		
Primary (1–7)	195	49.9
Ordinary level (S1–S4)	119	30.4
None	43	11.0
Advanced level (S5–S6)	27	6.9
Tertiary	7	1.8
Occupation		
Agriculture	291	74.4
Un-skilled manual	28	7.2
Skilled manual (vocational)	26	6.6
Sales services	17	4.3
Professional office work	15	3.8
Domestic service	9	2.3
Others	4	1.0
None	1	0.3
Ethnicity		
Iteso	303	77.5
Kumam	84	21.5
Others^a^	4	1.1

Data source—field findings from respondents

^a^Others = Acholi, Baganda and Basoga

### Malaria control measures by children under 5 years

A total of 779 children under 5 years who were resident in the households were included in the study. About half of them 400 (51.3%) were aged 3–4 years, with a mean age of 2.5 years (sd 1.2) and range of 3 years. A majority of the children 712 (91.4%) were using atleast one malaria preventive measure. The commonest malaria preventive measure used was bed nets 690 (96.9%) as in Additional file 2. This was confirmed by one KI from DHO’s office who said, “*The commonest and most effective preventive measure against malaria used by children under five years is sleeping under bed nets whether treated or untreated, although other measures such as closing doors or going to bed early and slashing bushes around homes exist*”.

Most children 617 (89.4%) were reported to have slept under bed nets the previous night to the survey, however only 392 (56.8%) slept under bed nets consistently (slept daily for the previous 12 months). A key informant from the DHO’s office noted that not all children were sleeping under bed nets consistently because of various reasons. He explained this saying, *“For example people in rural areas have few houses which are multi-purpose. The same house is a sitting room, store and sleeping room. People find it difficult to tie and un-tie bed nets on a daily basis, especially on nights visitors come and stay upto late. This defeats consistency in bed net use”.* A key informant from Stop Malaria Project agreed with the previous KI and said, *“We recently carried out a rapid assessment on bed net use in one nursery school in the Municipality and more than half of the children in Baby class said they never slept under the bed net the previous night”.*

A majority of these children 633 (91.3%) still used bed nets even if they slept on the floor/mat.

Most of the bed nets were obtained from NGOs (92.5%). Other sources of bed nets were government hospital, church/friend/relative, private hospital/clinic and from the VHT/Community leaders (Additional file 2). A key informant from DHO’s office confirmed this and said, “*Community members get bed nets from malaria control partners (NGOs) distributed through Ante-natal clinics in health centers as well as from the open market”*.

### Predisposing factors of respondents (knowledge about malaria)

A majority of the respondents 372/391 (95.1%) mentioned that malaria is transmitted by mosquitoes. However, some of the respondents among those who mentioned mosquitoes also had other incorrect views on malaria transmission including beliefs that malaria is transmitted by getting soaked in rain, eating bad/contaminated food and by drinking unboiled water (Additional file 3). The respondents had a fairly good knowledge about malaria control measures. The majority of them 364/391 (93.1%) correctly mentioned that malaria can be prevented by sleeping under a mosquito net. Other malaria preventive measures mentioned were clearing bushes around the home, destroying mosquito breeding sites, chemoprophylaxis, spraying with insecticides, use of mosquito repellants and closing door/windows early in the evening (Additional file 3).

Most respondents 307 (78.5%) reported that they had been sensitized about malaria transmission and control. The most common source of information was from radio programmes 197 (64.2%). Other sources of information mentioned were the church, community leaders and friends.

### Other respondent predisposing factors about consistent use of bed nets assessed

Most respondent 332/391 (84.8%) were aware of the fact that they were at risk of getting malaria and the majority 381 (97.4%) believed that consistent use of bed nets is beneficial because it prevents the user from getting malaria. Most respondents 298 (82.5%) had a positive attitude about using bed nets if they lacked raised beds or had their beddings on the floor. Respondents acknowledged that their chances of getting malaria were fairly high and only 64 (19.3%) believed that their chances of getting malaria were very low. Although a majority said bed nets were worth buying if not provided free of charge by government or NGOs, a few 56 (14.3%) believed that bed nets were harmful (Additional file 4).

A number of benefits of consistent use of bed nets were cited by respondents. These included reducing chances of getting malaria 287/391 (75.1%), reducing medical expenses 266 (69.6%) and relief from mosquito nuisances/painful bites 218 (57.1%).

Some respondents believed bed nets were harmful because they cause side effects including the itching and burning of the skin following use (ITNs). Furthermore, they believed that a bed net makes one feel stuffy and causes excessive heat/discomfort at night when one sleeps under it. A respondent from Gweri said, *“Much as most of us use bed nets, these nets sometimes cause itching of the skin/cancer, a lot of heat at night especially during dry season and they can even suffocate people at night”.*

Most respondents 336/391 (85.9%) acknowledged that mosquitoes are ever present in their sleeping rooms at night. A good number of them accordingly employ measures to protect themselves from mosquito bites. The most common protective measure used was sleeping under bed nets (94.8%) followed by burning mosquito coils and spraying houses with insecticide. This was confirmed by a KI from World Vision who said, “*Most people are nowadays using bed nets as the main malaria control measure since bed nets are distributed freely through ANC services and maternities by malaria control partners, especially if a household has more than one woman conceiving in a year”*.

Other malaria prevention measures employed were smoking the house by burning rubbish/dung, using local herbs and going to bed early. Most respondents (79.5%) said they slept under the bed net the previous night although only 44.3% slept under bed nets consistently (Additional file 4).

### Perceived enabling factors to consistent bed net use by under fives

A small proportion of children 89 (11.4%) were not sleeping under bed nets to protect them from getting malaria. Reasons for not sleeping under bed nets advanced by their caretakers included the high cost 71/89 (79.3%), fear that the children would suffocate 41 (46.6%) and to avoid heat and discomfort experienced when sleeping under bed nets 11 (12.1%).

The parents/caretakers of children often shared beds with children and their attitude towards bed nets influenced use of the bed nets by children. Respondents said the bed nets could be widely used consistently if they were adequately availed free of charge; while the colour, shape and size also affected consistent use. A few respondents 64/391 (17.7%) said the size of the bed nets was inappropriate and that they preferred very large bed nets. Another group 107 (29.6%) said the shape of the bed nets was inappropriate and they preferred conical bed nets that are easy to hang up their round houses.

This was confirmed by most key informants who said, *“The size, shape and multi-purpose nature of houses in rural areas can not facilitate use of common rectangular bed nets. It is difficult and tiresome to hang/unhang those bed nets on a daily basis; thus conical bed nets should be provided to rural residents if consistent use is to be enhanced”.*

Nearly half of respondents 196 (54.3%) disliked white bed nets and preferred mostly blue coloured bed nets. The need to use of bed nets was more during the rainy season when mosquito population was high. Most respondents 221 (38.8%) reported that they mainly used bed nets during rainy season when mosquitoes are many (Additional file 5). Most key informants agreed with this line of argument as one commented that, “*Many people quite believe that bed nets are used more during rainy season because that is when there are many mosquitoes because of much water within the homesteads”.*

### Results from key informant interviews

Most key informants mentioned bed nets as the main malaria control strategy in Soroti, followed by slashing compounds and closing doors early. *Other methods used included spraying houses with insecticides and destroying broken containers where mosquitoes could breed.*

Regarding source of bed nets for the people, *most of them mentioned NGOs, followed by government health centres and open market.*

Majority of key informants said the *major draw backs for consistent use of bed nets by the children under five years were the nature of sleeping rooms, nature of bed nets and cost of buying if not provided free of charge.* Others factors given were season of the year (dry) and presence of few mosquitoes in houses (Additional file 6).

### Factors influencing consistent use of bed nets among children under 5 years

#### Socio-demographic characteristics of respondents and children

Table 2 shows the association between socio-demographics characteristics of respondents and children and consistent use of bed nets by the children. Consistent use of bed nets was significantly higher among children whose caretakers did professional office work than their counterparts (OR: 0.25, 95% CI 0.10–0.67, p = 0.006). The age of children was a significant predictor of consistent bed net use in that the odds of consistent bed net use were 3 times higher among children aged 1–2 years than among those aged 3–4 years (Crude OR 2.945, 95% CI 1.321–3.559).

**Table 2:** Influence of socio-demographic characteristics of respondents and children on consistent use of bed nets by children under 5 years (N = 690)

Variable/Question	Consistent use of bed nets (n)		
	Yes (n = 392) n (%)	No (n = 298) n (%)	Un-adjusted OR (95% CI)
Age of respondent			
31–40	151 (57.6)	111 (42.4)	1.20 (0.48–98)
21–30	141 (60.5)	92 (39.5)	1.06 (0.42–2.66)
41–50	44 (42.3)	60 (57.7)	2.22 (0.85–5.80)
51–60	32 (59.3)	22 (40.7)	1.12 (0.40–3.14)
18–20	13 (61.9)	8 (38.1)	1.0
61+	11 (68.8)	5 (31.2)	0.74 (0.19–2.92)
Gender			
Male	223 (59.0)	155 (41.0)	1.22 (0.90–1.65)
Female	169 (54.2)	143 (45.8)	
Education level			
Primary (1–7)	191 (56.7)	146 (43.3)	0.76 (0.46–1.26)
O′ level	120 (56.6)	92 (43.4)	0.77 (0.45–1.30)
None	38 (50.0)	38 (50.0)	1.0
A′ level	33 (63.5)	19 (36.5)	0.58 (0.28–1.19)
Tertiary	10 (76.9)	3 (23.1)	0.30 (0.08–1.18)
Occupation			
Agriculture	276 (55.8)	219 (44.2)	1.0
Un-skilled manual	31 (55.4)	25 (44.6)	1.02 (0.58–1.77)
Skilled manual (vocational)	27 (52.9)	24 (47.1)	1.07 (0.60–1.93)
Sales services	19 (57.6)	14 (42.4)	0.93 (0.46–1.89)
Professional office work	25 (83.3)	5 (16.7)	0.25 (0.10–0.67)**
Domestic service	10 (58.8)	7 (41.2)	0.88 (0.33–2.36)
Others	4 (50.0)	4 (50.0)	1.58 (0.42–5.94)
Ethnicity			
Iteso	323 (60.0)	215 (40.0)	1.81 (1.26–2.60)**
Others	69 (45.4)	83 (54.6)	
Age of the children			
1–2 years	240 (69.8)	104 (30.2)	2.95 (2.15–4.03)**
3–4 years	152 (43.9)	194 (56.1)	

*P ≤ 0.1; **P < 0.05

The tribe of the respondent showed a significant association with consistent use of bed nets by children under 5 years in that the odds of consistent bed net use were higher among children of the Iteso than those of Kumam, Acholi, Baganda and Basoga combined (Crude OR = 1.81, 95% CI 1.26–2.60) but the difference was marginal. However, age, gender and education level of respondents did not show any statistically significant relationship with consistent use of bed nets by children under 5 years.

#### Influence of predisposing factors on children’ consistent use of bed nets

Table 3 shows the relationship between respondents’ predisposing factors and consistent use of bed nets by children under 5 years. The results show that there was no statistically significant relationship between knowledge about malaria transmission and control, perceived risk of malaria, having been sensitized about malaria control measures before, perceived benefits of bed nets, perceived harm and considering bed nets worth buying if not provided freely and consistent use by children under 5 years.

**Table 3:** Influence of respondents’ predisposing factors on consistent use of bed nets by children under 5 years (N = 690)

Variable/question	Consistent use of bed nets (n)		
	Yes/392 (%)	No/298 (%)	Un-adjusted OR (95% CI)
Knowledge on malaria transmission			
Knowledgeable (mosquitoes alone)	205 (58.2)	147 (41.8)	1.0
Less knowledgeable (also wrong ways of transmission mentioned)	170 (55.2)	138 (44.8)	1.13 (0.83–1.54)
No knowledge (don’t know)	17 (56.7)	13 (43.3)	1.07 (0.50–2.26)
Knowledge on malaria prevention			
Knowledgeable (> 2 correct responses)	169 (54.2)	143 (45.8)	0.82 (0.61–1.11)
Less knowledgeable (< 2 correct responses)	223 (59.0)	155 (41.0)	
Ever been sensitized about malaria control?			
Yes	315 (56.4)	244 (43.6)	0.91 (0.62–1.33)
No	77 (58.8)	54 (41.2)	
Think you are at risk of getting malaria?			
Yes	326 (55.5)	261 (44.5)	0.70 (0.45–1.08)
No	66 (64.1)	37 (35.9)	
Think consistent use of bed nets is beneficial?			
Yes	384 (57.1)	289 (42.9)	1.5 (0.57–3.92)
No	8 (47.1)	9 (52.9)	
Think bed nets are harmful in any way?			
Yes	55 (51.9)	51 (48.1)	0.79 (0.52–1.20)
No	337 (57.7)	247 (42.3)	
Think bed nets are worth buying if you had resources?			
Yes	374 (57.1)	281 (42.9)	1.26 (0.64–2.48)
No	18 (51.4)	17 (48.6)	

#### Influence of respondents’ enabling and need factors on children’s consistent bed net use

Table 4 shows the relationship between respondents’ enabling and need factors and consistent use of bed nets by the children. The results show that respondents’ consistent use of bed nets promoted consistent use of bed nets by their children by upto 37% (Crude OR 1.37, 95% CI 1.01–1.87). Likewise, children’s use of bed nets the previous night promoted consistent use of bed nets among them by 2 times (Crude OR 2.17, 95% CI 1.32–3.55).

**Table 4:** Influence of respondents’ enabling and need factors on children’s consistent use of bed nets (N = 690)

Variable/Question	Consistent use of bed nets (n)		
	Yes: n (%)	No: n (%)	Un-adj. OR (95% CI)
Presence of mosquitoes in houses			
Many	336 (55.7)	267 (44.3)	0.70 (0.44–1.11)
Some	56 (64.4)	31 (35.6)	
Respondents’ use of bed nets the previous night			
Yes	315 (58.0)	228 (42.0)	1.28 (0.87–1.88)
No	66 (52.0)	61 (480	
Respondent’s consistent use of bed nets			
Yes	188 (61.0)	120 (39.0)	1.37 (1.01–1.87)**
No	193 (53.3)	169 (46.7)	
Child’s use of bed net the previous night			
Yes	363 (58.8)	254 (41.2)	2.17 (1.32–3.56)**
No	29 (39.7)	44 (60.3)	
Respondent’s opinion regarding the size of bed net (n = 684)			
Inappropriate	84 (63.2)	49 (36.8)	1.37 (0.93–2.03)
Appropriate	306 (55.5)	245 (44.5)	
Opinion about the shape of bed nets			
Inappropriate	110 (53.4)	96 (46.6)	0.81 (0.58–1.13)
Appropriate	280 (58.6)	198 (41.4)	
Opinion about the colour of bed nets			
Inappropriate	205 (55.0)	168 (45.0)	0.83 (0.61–1.13)
Appropriate	185 (59.5)	126 (40.5)	
Season when the child uses the bed net			
Always	159 (56.6)	122 (43.4)	0.97 (0.71–1.32)
Rainy season	231 (57.3)	172 (42.7)	

*P ≤ 0.1, **p < 0.05

Respondents’ concern about the size of bed nets (that they preferred very large bed nets) promoted consistent use of bed nets by the children by 37% (Crude OR 1.373, 95% CI 0.929–2.028). Furthermore, there was no association between respondents’ perceived susceptibility, opinion about the shape, colour and season bed nets were used more and consistent use of bed nets by the children under 5 years.

#### Independent predisposing, enabling and need factors associated with consistent use of bed nets by children under 5 years

Table 5 shows the independent factors associated with consistent use of bed nets by children under 5 years. This formed the final model of the study, determined by Hosmer–Lemeshow-fitness test, with a p-value of 0.578 and − 2loglikelihood of 830.808.

**Table 5:** Final logistic regression model for factors influencing consistent use of bed nets by under fives in Soroti district, North Eastern Uganda

Variable	Consistent bed net use (n = 670) (N = 670) (N = 670)		Unadjusted OR/95%CI	Adjusted OR 95% CI
	Yes/381 (%)	No/289 (%)		
Occupation				
Agriculture	276 (72.4)	219 (75.8)	1.0	1.0
Domestic service	10 (02.6)	07 (02.4)	0.88 (0.33–2.36)	0.70 (0.24–2.02)
Un-skilled manual	31 (08.1)	25 (08.7)	1.02 (0.58–1.77)	0.86 (0.45–1.60)
Skilled manual	27 (07.1)	23 (08.0)	1.07 (0.60–1.93)	1.38 (0.74–2.59)
Sales services	19 (05.0)	14 (04.8)	0.93 (0.46–1.89)	0.94 (0.43–2.02)
Professional office work	25 (06.6)	05 (01.7)	0.25 (0.10–0.67)**	0.21 (0.08–0.58)**
Others	04 (01.0)	04 (01.4)	1.58 (0.42–5.94)	1.38 (0.34–5.59)
Knowledge on malaria prevention	169 (44.4)	143 (49.5)	0.82 (0.61–1.11)	0.78 (0.55–1.11)
Age of children (1–2 years)	240 (63.0)	104 (36.0)	2.95 (2.15–4.03)***	3.15 (2.26–4.39)***
Child’s use of bed net previous night	363 (95.3)	254 (87.9)	2.17 (1.32–0.56)**	1.93 (1.11–3.36)**
Respondent’ consistent bed net use	188 (49.3)	120 (41.5)	1.37 (1.01–1.87)**	1.17 (0.84–1.63)
Perceived susceptibility (many)	336 (88.2)	267 (92.4)	0.7 (0.44–1.11)	0.55 (0.33–0.93)**
Perceived risk of getting malaria	326 (85.6)	261 (90.3)	0.70 (0.45–1.08)	0.57 (0.35–0.92)**
Opinion on bed net size	84 (22.0)	49 (17.0)	1.37 (0.93–2.03)	1.87 (1.07–3.28)**
Opinion about shape of bed net	110 (28.9)	96 (33.2)	0.81 (0.58–1.13)	0.58 (0.36–0.94)**

***P < 0.001, **P < 0.05, *P ≤ 0.1

The final logistic regression model used was;

Logit P (Child used bed net consistently) = − 1.6 + 1.14 Age of child + 0.7 Child’s use of bed net previous night + 0.7 Opinion on size of bed net − (1.4 Occupation-professionals + 0.6 perceived susceptibility + 0.5 no perceived risk of getting malaria + 0.5 Opinion on rectangular shape of bed net).

The independent factors associated with consistent use of bed nets by children under 5 years were categorized under predisposing, enabling and need factors.

- **Pre-disposing factors** in the final model, consistent use of bed nets was influenced by occupation of respondent, age of children, childrens’ use of bed nets the previous night to the survey, respondent’s perceptions that mosquitoes were few in sleeping rooms and being at risk of getting malaria. The results show that the odds of consistent use of bed nets were 79% higher among children of professionals than among children of respondents who practiced agriculture (Adj OR: 0.21, 95% CI 0.08–0.58, p = 0.003). On the other hand, the odds of consistent bed net use were 3 times higher among children aged 1–2 years than among the older children (Adj OR: 3.15; 95% CI 2.26–4.39, P = 0.000). As expected, children who used bed nets the previous night to the survey were more likely to use them consistently (Adj OR; 1.93 95% CI 1.11–3.36, P = 0.02). Furthermore, respondent’s perceived risk of getting malaria influenced their children’s consistent use of bed nets in that the odds of consistent use of bed nets were 43% lower among children of respondents who said they were not at risk of getting malaria than among those of their counter parts (Adj OR: 0.57, 95% CI 0.35–0.92, P = 0.02).
- **Enabling factors** The final model shows that respondents’ opinions about bed net size and shape influenced consistent use of bed nets by the children. The results show that children of respondents who preferred very large bed nets were more likely to use bed nets consistently than those of respondents who preferred medium sized bed nets (Adj OR: 1.87, 95% CI 1.07–3.28, p = 0.03). Likewise, children of respondents who said they preferred conical bed nets were more likely to use bed nets consistently than children of respondents who said they preferred rectangular bed nets (Adj OR 0.58; 95% CI 0.36–0.94, p = 0.03).
- **Need factors** The final model shows that children of respondents who were aware that many mosquitoes are ever present in houses were more likely to use bed nets consistently than those of their counter parts. The results show that the odds of consistent use of bed nets were 45% lower among children whose caretakers said the mosquitoes were few in houses than among those of their counter parts (Adj OR: 0.55, 95% CI 0.33–0.93, p = 0.02).

## Discussion

### Use of bed nets by children under 5 years

The results show that the prevalence of consistent use of bed nets by children under 5 years for prevention of malaria in Soroti district was 56.8%. This prevalence is low compared to the WHO recommended coverage levels of 85%. The definition for consistent use of nets was stringent and the prevalence could have been underestimated due to recall bias. The reasons for inconsistent use of bed nets by children were multiple, including torn/worn out bed nets, frequent changing of sleeping spaces due to lack of space on some days. This finding was re-affirmed by most KI when they said the multi-purpose nature of the small-few houses in rural areas could not support consistent use of bed nets by the children.

This proportion was slightly higher than that described among care-givers of children under 10 years in Central Malaita, Solomon Islands where 52% of the respondents use bed nets consistently [36] and lower than that found among respondents in Ethiopia where the majority of respondents (85.7%) used nets daily [37].

The results show that younger children (1–2 years) used bed nets more consistently than older ones, inline with findings by a study done to evaluate bed net use in Africa [38]. This can be explained by the fact that older children are less likely to use bed nets as priority is given to younger ones, especially if the bed nets are as few as one in the household. Another reason could be that older children experience lower episodes of malaria and the caretakers perceive that they are protected from malaria. This is a negative finding because all children are vulnerable to malaria attack and should be equally protected.

This finding is also in line with other studies done in other parts of the world pertaining factors influencing the use of bed nets by children under 5 years [1–5, 7–16].

The proportion of children who used the bed net the previous night to the survey was high 89.4%, a figure that is far much higher than the national estimates, which show a slight increase in bed net use the previous night from 41% in 2009 [14] to 53% in 2011 [39]. The main reason for the high usage rates in Soroti was that several malaria control partners were present in the district from the time of the Lord’s Resistance Army (LRA) insurgency and bed nets were distributed to residents free of charge.

Reasons for children not sleeping under the bed net the previous night included bed nets not having dried after being washed, torn nets and nets still being repaired.

These findings are consistent with findings within Uganda where the most common reason cited for non-usage of bed nets the previous night in 2009 was that the net was not hung (58% of households) and in Mid-Eastern region where Soroti falls, the reasons included net not hang (65.1%) and that nets were too old/had many holes that could not be repaired (9.3%) [14]. Similar reasons were identified in a systematic review in Africa where nets being damaged was among the reasons for children not using bed nets the previous night [40].

The main reason for non use of bed nets by children under 5 years was that bed nets were expensive and difficult to come-by. Similar results were reported in Nigeria where some of the factors associated with use of LLINs included the cost of ITNs followed by their non-availability [41]. Several other studies conducted on the use of bed nets among children under 5 years and other age groups confirm that a number of factors are attributed to low usage of bed nets by their populace [18–25]

### Pre-disposing factors

Generally, 47.7% of respondents were knowledgeable about malaria. This proportion was low and could have contributed to the low consistent use of bed nets by children under 5 years. This findings relate to those from Tanzania where knowledge was found to affect ITN uptake/use [42]. Much as most respondents ably mentioned that malaria is transmitted by mosquitoes, quite a good number believed that other factors could cause malaria, including eating contaminated food, drinking unboiled water and being beaten by rain/coldness. Similar observations were made in a study done in rural Burkina Faso where it was established that other aetiological factors such as humidity, exposure to rain and cold were widely being held as causative factors of malaria in addition to mosquitoes [43].

The findings were also consistent with findings observed in Northern Ghana, where local community knowledge about malaria played a vital role in ensuring consistent use of ITNs. The study found out that much as people recognized the term malaria, they had limited biomedical knowledge of the disease- including its etiology, the role of the vector and host response. This could have amounted to failure of the community members to recognize the role of ITNs in malaria prevention as most of them said they were for nuisance reduction [44]. The findings further relate to those in Makueni district, Kenya where use of ITNs in households was positively associated with awareness about ITNs, marital status and occupation [45].

A lot of sensitization was being done on radio by sponsored VHT members on ensuring that children stay healthy and free from diseases such as malaria by using bed nets, maintaining good hygiene and taking children early for treatment. Despite presence of health promotion activities in the district, some respondents (22%) said they had never been sensitized about malaria prevention and control. This could be because they did not own radios which was the main source of information on malaria or they did not believe in health education messages on radio to be good enough for adoption. This could imply that the DHO’s office needs to design a health worker house-to-house health education program on crucial health issues, such as malaria control.

These findings were in line with a study done in the Democratic Republic of Congo where lack of health education was found to be the most important factor affecting bed net use in the villages outside Kinshasa. Development of an educational programme, particularly one directed towards parents, was necessary to reduce misconceptions and increase prevalence of bed net use among all age groups [46].

Respondents’ perceived risk of getting malaria is essential in promoting bed net use. This is because people always adopt positive preventive practices such as consistent bed net use basing on what they believe. This could explain why risk of getting malaria remained significant in the final logistic regression model, as the odds of consistent use were 64% lower among children of respondents who never perceived to be at risk of getting malaria.

Among those who used bed nets, protection against mosquito nuisances/painful bites was given as a major reason for using bed nets (54.1%) instead of repelling and killing mosquitoes that transmit malaria. Since the respondents did not know the life-threatening danger of mosquito bites (malaria) they could not be expected to ensure their children sleep under bed nets consistently. These findings relate to those of a study done in Central Malaita, Solomon Islands where 59% of the respondents said that the main reason for use of bed nets was protection against mosquito nuisances/painful bites [36].

In other related studies, there were several predisposing factors linked to none use of ITNs by children under five and other age groups. However, lack of knowledge about malaria transmission by mosquitoes and prevention using ITNs was among the biggest gaps identified in some of the studies [26, 28, 29, 31, 33].

### Enabling factors

Reported consistent bed net use among under fives was only 56.8%. This could be because of barriers to consistent bed net use which were significant in the final logistic regression model. The respondents’ opinions about the bed net medium size and rectangular shape contributed to the low bed net usage rates among their children. The area being rural and residents dominantly sleeping in small-round houses did not facilitate use of rectangular bed nets which were commonly supplied by malaria control partners in the district.

Similar barriers were observed in Ethiopia where respondents said rectangular bed nets were difficult to hang and unhang every day in the round houses they slept in and they preferred conical bed nets which are easy to hang [47]. This was re-echoed by most key informants interviewed when they said consistent bed net use could be enhanced in rural areas by providing larger conical bed nets which could easily be tied up the round huts during day and spread on beddings at night.

For the children under 5 years who were reported not to use bed nets at all, several reasons were fronted by their caretakers including the high cost of bed nets, lack of free nets (missed during free distribution), fear of discomfort and heat experienced when sleeping under bed nets. The issue of excessive heat was more prominent during the dry season when the sun gets scotching hot and residual heat is felt in houses at night.

Similar observations were made in a study done by WHO to monitor and evaluate bed net possession and use by children under 5 years in Africa, where it was found out that not all bed nets owned were being used and major factors were that nets were scarce, nets use depended on the season (less during dry-hot weather) and that not all nets in the households were used by young children as a priority group to be protected from malaria [15]. Several other studies revealed that factors influencing the use of bed nets in children under five were related to size of the house, shape and size of the net as well as other environmental factors [34, 37, 39–42]. This implies the size of bed nets needs to be increased as respondents said they would prefer very large bed nets, such as 6 * 6 m or 5 * 6 m; and conical bed nets provided if consistent use is to be enhanced.

### Need factors

Perceived susceptibility to malaria attack is essential in compelling an individual to adopt prevention mechanisms in place. This was the reason ability to see many mosquitoes remained significant in the final logistic regression model of the study. The findings showed that children of respondents who had knowledge of presence of many mosquitoes in their houses always were more likely to use bed nets consistently than those of their counter parts. The few respondents (14%) who said the mosquitoes were few could have contributed to the low consistent use of bed nets among children since they could have been swayed away with the idea that the malaria transmitters were few.

Similar findings were observed in Zimbabwe, where among other reasons for not purchasing LLINs as priority were not liking to use nets; not knowing the efficacy of the nets; not seeing many mosquitoes around their homes [47].

The results in this paper should be interpreted in light of the following limitations; Re-call bias due to the long 1-year period, leading to reduction in the observed outcome variable. Problem of classification bias because one respondent could have one child using nets consistently while others were not. Attitudes were not adequately measured due to a limitation of the questionnaires used and the study being a survey could only show associations and not causal pathways.

## Conclusions

The study revealed that most of the respondents were aware of malaria transmission, prevention and control measures. However, consistent use of bed nets by children under 5 years was still low, much lower than the WHO/NMCP target of 85 percent. Consistent use of bed nets by children under 5 years in Soroti district depended mainly on the age of the child; child’s use the previous night, respondent’s occupation, perceived susceptibility, perceived risk of getting malaria, size and shape of the bed net. The community members perceived that 1–2-year-old children were more vulnerable to malaria attack thus should sleep under bed nets more consistently than older under fives. Therefore, malaria control partners should consider providing larger conical bed nets in rural areas to enhance their consistent use. The government should consider establishing an active house-to-house malaria control (health education) programme using community health workers to demonstrate ways that enhance consistent bed net use amongst under fives. The key messages should emphasize the ability of one infected mosquito to transmit malaria to the whole household and the effectiveness of insecticide-treated bed nets in reducing transmission. Ministry of Health should embark on community sensitization using appropriate media houses and communication channels to protect all under fives from mosquitoes and malaria. Finally, the WHO should carry out studies emphasizing on determination of the effect of attitudes on consistent bed net use.

## Supplementary Information

**Additional file 1.** Assessment of respondents’ knowledge about malaria.

**Additional file 2.** Table showing malaria control measures used by children under 5 years in Soroti district.

**Additional file 3.** Table showing predisposing factors (knowledge about malaria).

**Additional file 4.** Table showing other predisposing factors about consistent use of bed nets assessed.

**Additional file 5.** Table showing enabling factors to consistent use of bed nets.

**Additional file 6.** Table showing results from key informants.

## Data Availability

Datasets used in the analysis are available from the corresponding author on reasonable request.

## Abbreviations

ADHO: Assistant District Health Officer
AMREF: African Medical Research and Education Foundation
BRAC: Bangladesh Rural Advancement Committee
DHO: District Health Office
DHE: District Health Educator
GTZ: German Society for Technical Cooperation
HH: Household
IMA: International Mid-Wife Assistance
IRB: Institutional Review Board
IRS: Indoor Residual Spraying
ITN: Insecticide Treated Nets
KAPs: Knowledge, Attitudes and Practices
KI: Key Informant
LLINs: Long-Lasting Insecticidal Nets
MCH: Maternal and Child Health
MCP: Malaria Control Programme
MoH: Ministry of Health
NGO: Non-Governmental Organization
PMI: Presidential Malaria Initiative
RBM: Roll Back Malaria
TSMP: Teso safe Motherhood Project
UBOS: Uganda Bureau of Statistics
UNCST: Uganda National Council of Science and Technology
UNICEF: United Nations International Children’s Fund
UNMCP: Uganda National Malaria Control Programme
USAID: United States Agency for International Development
WHA: World Health Assembly
WHO: World Health Organization
