# Supplementary material for: Factors influencing consistent use of bed nets for the control of malaria among children under 5 years in Soroti District, North Eastern Uganda
Source: Malar J. 2022 Dec 2;21:363. doi: 10.1186/s12936-022-04396-z (PMC9716664; doi:10.1186/s12936-022-04396-z)
Supplement: Supplementary file 1 — Additional file 1. Assessment of respondents’ knowledge about malaria. [file 12936_2022_4396_MOESM1_ESM.docx]

**Assessment of respondents’ knowledge about malaria**

At analysis, two (2) areas (transmission & prevention) were used to assess respondents’ knowledge of malaria, following a procedure adopted from a study done by Nganda, et al (2004) in Tanzania. Knowledge on malaria transmission was categorized into three: 1. Knowledgeable**:** if respondent mentioned infected mosquito bites as the only means of malaria transmission. 2. Less knowledgeable**:** if respondent mentioned other wrong ways of malaria transmission in addition to infected mosquito bites. 2. No knowledge**:** - if respondent mentioned only wrong ways of malaria transmission or gave the “I don’t know” response.

A respondent was considered to be knowledgeable about malaria prevention if he/she mentioned at least 2 of the four effective control measures against malaria (use of bed nets, spraying with insecticides, chemoprophylaxis and use of repellants).

Respondents who mentioned one or none of the four effective measures were considered less knowledgeable. The mean knowledge score was obtained by determining the mean of the two proportions of respondents who were knowledgeable in malaria transmission and prevention.

Univariate analysis was done by summarizing variables using frequencies and proportions, shown in frequency tables. Descriptive statistics such as means, mode and standard deviations were used to summarize continuous variables while categorical variables were summarized with frequencies and proportions.

At Bivariate analysis, each independent variable in each objective was investigated singly with the outcome variable using logistic regression to determine Odd’s Ratios (ORs) and respective p-values.

At multi-variable analysis, a significance level of P≤0.1 was used to determine independent variables for further analysis. Other variables analyzed were determined from **biological plausibility and literature**. The variables were entered into a Binary multivariable logistic regression model to predict associations between the dependent variable **“children’s consistent use of bed nets” and the independent variables**.

The final logistic regression model was obtained using a step-wise forward approach and Hosmer-Lemeshow-goodness-of-fit test. The final model was based on Hosmer-Lemeshow-fitness test p-value of 0.578 and -2loglikelihood of 830.808.

All the significant predisposing, enabling and need factors were then reported using their ORs, 95 % Confidence Intervals (CI) as well as P-values. P-values less than 0.05 were considered to show statistically significant associations between consistent use of bed nets and the independent variables.

Interpretations were written out to bring out meaning for the readers to appreciate the relationships between different independent variables and the consistent use of mosquito nets by children under five years in Soroti district.

For qualitative data from Key Informant interviews, short notes were expanded. Data was organized and sorted into different themes emerging from the responses to questions asked in line with the objectives of the study. Relevant findings were presented in a table. Results were then written out by summarizing, directly quoting and putting them to reinforce quantitative findings.
