# Supplementary material for: Factors influencing consistent use of bed nets for the control of malaria among children under 5 years in Soroti District, North Eastern Uganda
Source: Malar J. 2022 Dec 2;21:363. doi: 10.1186/s12936-022-04396-z (PMC9716664; doi:10.1186/s12936-022-04396-z)
Supplement: Supplementary file 2 — Additional file 2. Table showing malaria control measures used by children under 5 years in Soroti district. [file 12936_2022_4396_MOESM2_ESM.docx]

## Table showing malaria control measures used by children under five years in Soroti district

| Variable | Frequency (n=779) | Percentages (%) |
| --- | --- | --- |
| **Age of child** | | |
| 3-4 years | 400 | 51.3 |
| 1-2 years | 379 | 48.7 |
| **Child currently using protection against malaria?** | | |
| Yes | 712 | 91.4 |
| No | 67 | 8.6 |
| **Protective measures currently used (n=712)** | | |
| Bed net | 690 | 96.9 |
| Coils | 93 | 13.1 |
| Sprays | 81 | 11.4 |
| Smoke from burning rubbish/dung | 53 | 07.4 |
| Herbs | 17 | 02.4 |
| Others | 5 | 0.01 |
| **Child slept under a bed net the previous night to survey (n=690)** | | |
| Yes | 617 | 89.4 |
| No | 73 | 10.6 |
| **Consistent use of bed nets (use daily for the past 12 months)** | | |
| Yes | 392 | 56.8 |
| No | 298 | 43.2 |
| **What if child sleeps on floor/mat, still uses bednet?** | | |
| Yes | 633 | 91.3 |
| No | 57 | 08.7 |
| **Source of child’s bed net (Multiple responses, n=690)** | | |
| NGO | 638 | 92.5 |
| Government HC | 587 | 85.1 |
| Open market | 528 | 76.5 |
| Government Hospital | 172 | 24.9 |
| Church, friend/relative | 126 | 18.2 |
| Private Hospital/clinic | 68 | 9.9 |
| VHT/Community leader | 55 | 8.0 |
| Hawkers | 36 | 5.2 |
| Private pharmacy | 23 | 3.3 |

*Data source - field findings from respondents*
