# Supplementary material for: Factors influencing consistent use of bed nets for the control of malaria among children under 5 years in Soroti District, North Eastern Uganda
Source: Malar J. 2022 Dec 2;21:363. doi: 10.1186/s12936-022-04396-z (PMC9716664; doi:10.1186/s12936-022-04396-z)
Supplement: Supplementary file 3 — Additional file 3. Table showing predisposing factors (knowledge about malaria). [file 12936_2022_4396_MOESM3_ESM.docx]

## Table showing predisposing factors (knowledge about malaria)

| Variable | Frequencies (n=391) | Percentages (%) |
| --- | --- | --- |
| **How malaria is transmitted (Multiple responses)** | | |
| By mosquitoes | 372 | 95.1 |
| Getting soaked in rain | 118 | 30.2 |
| Drinking unboiled water | 40 | 10.2 |
| Eating bad or contaminated food | 40 | 10.2 |
| Others | 16 | 4.1 |
| I don't Know | 9 | 2.3 |
| Direct contact with infected persons | 6 | 1.5 |
| **Malaria preventtion measures respondents are aware of** | | |
| Sleep under mosquito net | 364 | 93.1 |
| Clear bushy areas around the home | 178 | 45.5 |
| Destroy mosquito breeding sites | 168 | 43.0 |
| Chemical prophylaxis | 103 | 26.3 |
| Close doors and windows early in the evening | 97 | 24.8 |
| Spraying the house with insecticide | 69 | 17.6 |
| Use of mosquito screens on vents, windows and doors | 34 | 8.7 |
| Use of mosquito repellants | 34 | 8.7 |
| I don’t know | 7 | 1.8 |
| Others | 3 | 0.8 |
| **Ever been sensitized about malaria control measures** | | |
| Yes | 307 | 78.5 |
| No | 84 | 21.5 |
| **Source of information about malaria control (Multiple responses, n=307)** | | |
| Radio | 197 | 64.2 |
| NGOs | 170 | 55.4 |
| Health worker | 142 | 46.3 |
| Church | 84 | 27.4 |
| Community leader | 61 | 19.9 |
| Friends | 52 | 16.9 |
| Family | 46 | 15.0 |
| Prints/newspapers | 36 | 11.7 |
| Others | 11 | 3.6 |

*Data source - field findings from respondents*
