# Supplementary material for: Factors influencing consistent use of bed nets for the control of malaria among children under 5 years in Soroti District, North Eastern Uganda
Source: Malar J. 2022 Dec 2;21:363. doi: 10.1186/s12936-022-04396-z (PMC9716664; doi:10.1186/s12936-022-04396-z)
Supplement: Supplementary file 4 — Additional file 4. Table showing other predisposing factors about consistent use of bed nets assessed. [file 12936_2022_4396_MOESM4_ESM.docx]

## Table showing other predisposing factors about consistent use of bed nets assessed

| Variable | Frequencies (n=391) | Percentages (%) |
| --- | --- | --- |
| **Sleep under bed net if beddings are on the floor/mat (n=361)** | | |
| Yes | 298 | 82.5 |
| **Feel at risk of getting malaria?** |  |  |
| Yes | 332 | 84.9 |
| **Respondents rating of their chances of getting malaria** | | |
| Moderate | 198 | 59.6 |
| Very high | 70 | 21.1 |
| Small | 64 | 19.3 |
| **Do you think consistent use of bed nets is beneficial?** | | |
| Yes | 381 | 97.4 |
| **Do you think bed nets are harmful in any way?** | | |
| Yes | 56 | 14.3 |
| **Think bed nets are worth buying if not provided by Government/NGOs?** | | |
| Yes | 363 | 92.8 |
| **Benefits of consistent use of bed nets (Multiple responses, n=381)** | | |
| Reduces chances of getting malaria | 287 | 75.1 |
| Reduce medical expenses | 266 | 69.6 |
| It reduces nuisances (painful bites) of mosquitoes | 218 | 57.1 |
| Reduces nuisances from other crawling insects | 106 | 27.7 |
| Keeps people warm at night | 29 | 7.6 |
| Others | 6 | 1.6 |
| **Harmful effects of bed nets (Multiple responses**, **n=56**) | | |
| They cause side effects like itching, burning of skin | 44 | 80.0 |
| The bed net generates heat at night and causes discomfort | 26 | 47.3 |
| They can suffocate children/pregnant women | 11 | 20.0 |
| Chemicals pollute the environment | 3 | 5.5 |
| Chemicals used to treat nets cause cancer | 1 | 1.8 |
| **Presence of mosquitoes in houses** | | |
| Many | 336 | 85.9 |
| Few | 55 | 14.1 |
| **Currently using anything for protection against mosquito bites?** | | |
| Yes | 381 | 97.4 |
| **Malaria protective measures used by respondent (Multiple responses, n=381)** | | |
| Bed nets | 361 | 94.8 |
| Coils | 91 | 23.9 |
| Sprays | 84 | 22.0 |
| Smoke from burning dung/rubbish | 33 | 8.7 |
| Herbs | 14 | 3.7 |
| Others | 1 | 0.3 |
| **Use of bed net the previous night (n=361)** | | |
| Yes | 287 | 79.5 |
| **Respondents’ consistent use of bed nets** | | |
| Yes | 160 | 44.3 |

*Data source - field findings from respondents*
