# Supplementary material for: Factors influencing consistent use of bed nets for the control of malaria among children under 5 years in Soroti District, North Eastern Uganda
Source: Malar J. 2022 Dec 2;21:363. doi: 10.1186/s12936-022-04396-z (PMC9716664; doi:10.1186/s12936-022-04396-z)
Supplement: Supplementary file 5 — Additional file 5. Table showing enabling factors to consistent use of bed nets. [file 12936_2022_4396_MOESM5_ESM.docx]

## Table showing enabling factors to consistent use of bed nets

| Variable | Frequencies (n=361) | Percentages (%) |
| --- | --- | --- |
| **Reasons for child not using bed nets (Multiple responses, n=89)** | | |
| They are expensive | 71 | 79.3 |
| They can suffocate children/pregnant women | 41 | 46.6 |
| The bed net generates heat at night and causes discomfort | 11 | 12.1 |
| Chemicals pollute the environment | 2 | 1.7 |
| **Opinions about the white, medium and rectangular bed nets being used** | | |
| **(i) Opinion about size** | | |
| Inappropriate size | 64 | 17.7 |
| **ii) Opinion about shape** | | |
| Inappropriate shape | 107 | 29.6 |
| **iii) Opinion about colour** | | |
| Inappropriate colour | 196 | 54.3 |
| **iv) Season when bed nets are mostly used** | | |
| Rainy season | 221 | 61.2 |
| Always | 140 | 38.8 |

*Data source - field findings from respondents*
