# Supplementary material for: Factors influencing consistent use of bed nets for the control of malaria among children under 5 years in Soroti District, North Eastern Uganda
Source: Malar J. 2022 Dec 2;21:363. doi: 10.1186/s12936-022-04396-z (PMC9716664; doi:10.1186/s12936-022-04396-z)
Supplement: Supplementary file 6 — Additional file 6. Table showing results from key informants. [file 12936_2022_4396_MOESM6_ESM.docx]

**Table showing results from Key Informants**

| Variable | Responses |
| --- | --- |
| **Malaria preventive measures used in Soroti** | Mosquito bed nets |
|  | Slashing the compound |
|  | Closing doors early |
|  | Spraying with insecticides |
|  | Destroying broken containers in homes |
|  |  |
| **Source of bed nets** | NGOs |
|  | Government health centres |
|  | Open market |
|  |  |
| **Factors affecting consistent bed net use** | Nature of houses (small, round and multi-purpose) |
|  | Nature of bed nets (rectangular and medium) |
|  | Cost of buying is high for rural people |
|  | Season of the year |
|  | Presence of few mosquitoes |

*Data source - field findings from key informants*
